# Characterization of an Unusual Severe Yellow Fever Outbreak in the Colombian Andean Region

**DOI:** 10.1093/ofid/ofag636

**Published:** 2026-10-21

**Authors:** Alex Julián Forero-Delgadillo, Jeison Andrés Morales-Olivera, Paula O Narvaez-Ramirez, Natalia Sanabria-Herrera, Valeria Aguirre Gutierrez, Lina F Martinez-Lemus, Cristian C Serrano-Mayorga, Diana Catalina Ramírez-Reyes, Juan Diego Melo-Pedraza, Julián Celis-Guzmán, Gustavo González-Varona, Henry Robayo-Amortegui, Jessica María Forero, Erwin Hernandez, Ingrid G Bustos, Sara Duque, Esteban Garcia-Gallo, Luis F Reyes

**Affiliations:** Unisabana Center for Translational Science, School of Medicine, Universidad de La Sabana, Chía, Colombia; Clinical and Translational Medicine Research Group, Critical Physiology Subdivision, Intensive Care Department, Hospital Federico Lleras Acosta, Ibagué, Colombia; School of Medicine, Faculty of Health Sciences, Universidad del Tolima, Ibagué, Colombia; Biosciences PhD Program, School of Engineering, Universidad de La Sabana, Chía, Colombia; Department of Critical Care Medicine, Clínica Keralty, Ibagué, Colombia; Unisabana Center for Translational Science, School of Medicine, Universidad de La Sabana, Chía, Colombia; Clinical and Translational Medicine Research Group, Critical Physiology Subdivision, Intensive Care Department, Hospital Federico Lleras Acosta, Ibagué, Colombia; School of Medicine, Faculty of Health Sciences, Universidad del Tolima, Ibagué, Colombia; Department of Critical Care Medicine, Clínica Keralty, Ibagué, Colombia; Doctorado en Ciencias Clínicas, Facultad de Medicina, Universidad de La Sabana, Chía, Colombia; School of Medicine, Universidad Salud Colombia, Ibagué, Colombia; Unisabana Center for Translational Science, School of Medicine, Universidad de La Sabana, Chía, Colombia; Department of Critical Care Medicine, Clínica Universidad de La Sabana, Chía, Colombia; Unisabana Center for Translational Science, School of Medicine, Universidad de La Sabana, Chía, Colombia; Department of Critical Care Medicine, Clínica Universidad de La Sabana, Chía, Colombia; Unisabana Center for Translational Science, School of Medicine, Universidad de La Sabana, Chía, Colombia; Department of Critical Care Medicine, Clínica Universidad de La Sabana, Chía, Colombia; Unisabana Center for Translational Science, School of Medicine, Universidad de La Sabana, Chía, Colombia; Unisabana Center for Translational Science, School of Medicine, Universidad de La Sabana, Chía, Colombia; Biosciences PhD Program, School of Engineering, Universidad de La Sabana, Chía, Colombia; Clinical and Translational Medicine Research Group, Critical Physiology Subdivision, Intensive Care Department, Hospital Federico Lleras Acosta, Ibagué, Colombia; School of Medicine, Faculty of Health Sciences, Universidad del Tolima, Ibagué, Colombia; Clinical and Translational Medicine Research Group, Critical Physiology Subdivision, Intensive Care Department, Hospital Federico Lleras Acosta, Ibagué, Colombia; School of Medicine, Faculty of Health Sciences, Universidad del Tolima, Ibagué, Colombia; Clinical and Translational Medicine Research Group, Critical Physiology Subdivision, Intensive Care Department, Hospital Federico Lleras Acosta, Ibagué, Colombia; School of Medicine, Faculty of Health Sciences, Universidad del Tolima, Ibagué, Colombia; Department of Critical Care Medicine, Clínica Keralty, Ibagué, Colombia; Clinical and Translational Medicine Research Group, Critical Physiology Subdivision, Intensive Care Department, Hospital Federico Lleras Acosta, Ibagué, Colombia; Department of Critical Care Medicine, Clínica Keralty, Ibagué, Colombia; Doctorado en Ciencias Clínicas, Facultad de Medicina, Universidad de La Sabana, Chía, Colombia; Department of Critical Care Medicine, Extracorporeal Life Support Unit, Fundación Clínica Shaio, Bogotá, DC, Colombia; Department of Pediatric Nephrology, Fundación Valle del Lili, Cali, Colombia; Unisabana Center for Translational Science, School of Medicine, Universidad de La Sabana, Chía, Colombia; Unisabana Center for Translational Science, School of Medicine, Universidad de La Sabana, Chía, Colombia; ISARIC, Pandemic Sciences Institute, University of Oxford, Oxford, UK; ISARIC, Pandemic Sciences Institute, University of Oxford, Oxford, UK; Unisabana Center for Translational Science, School of Medicine, Universidad de La Sabana, Chía, Colombia; Department of Critical Care Medicine, Clínica Universidad de La Sabana, Chía, Colombia; ISARIC, Pandemic Sciences Institute, University of Oxford, Oxford, UK

**Keywords:** Colombia, critical illness, intensive care, outbreak, yellow fever

## Abstract

**Background:**

Yellow fever (YF) reemerged in Colombia’s Andean region during 2024–2025, outside the country’s historically recognized endemic areas. We characterized critically ill adults admitted during this outbreak and described clinical features, organ dysfunction, supportive care, and in-hospital outcomes.

**Methods:**

We conducted a prospective observational cohort study of consecutive adults with reverse transcription polymerase chain reaction (RT-PCR)–confirmed YF admitted to the national referral intensive care unit in Ibagué, Tolima, Colombia, from March through September 2025. Clinical, laboratory, complication, intervention, and outcome data were prospectively captured using a standardized case report form.

**Results:**

Sixty-one patients were analyzed; 22 died (36.1%). Non-survivors had lower platelet counts and fibrinogen and higher INR, aminotransferases, lactate dehydrogenase, and creatinine at ICU admission. Shock, acute kidney injury, acute respiratory distress syndrome, disseminated intravascular coagulation, and severe bleeding were more frequent among non-survivors. Prior YF vaccination was self-reported by 10 patients (16.4%).

**Conclusions:**

Severe YF in this Andean outbreak was characterized by hepatic-coagulation abnormalities and multisystem organ dysfunction. The occurrence of critical illness outside historically recognized endemic areas supports surveillance and vaccination strategies that can adapt to changing epidemiological risk.

Yellow fever (YF) is a life-threatening hemorrhagic arboviral disease transmitted by *Aedes*, *Haemagogus*, and *Sabethes* mosquitoes, with a fatality rate of approximately 67% among severe icteric cases according to international cohorts [1]. Nearly 900 million individuals remain at risk across endemic regions of Africa and the Americas [2], and recent years have been marked by renewed viral circulation and geographic expansion [3, 4]. In 2024, 61 cases were confirmed in the Americas [5], increasing to 235 cases and 96 deaths by 2025, prompting regional alerts and updates from the Pan American Health Organization (PAHO) and World Health Organization (WHO) [6, 7]. Notably, cases have emerged outside historically endemic Amazonian regions, including densely populated areas such as the state of São Paulo, Brazil, and Tolima, Colombia [6, 8]. This epidemiological shift raises critical questions regarding the clinical phenotype, health-system burden, and impact of severe YF in newly affected regions.

The geographic spread of YF out of the Amazon basin appears to be driven by a confluence of environmental and human-induced factors that are reshaping transmission risk among vulnerable populations [9, 10]. Climate variability, including changes in precipitation patterns and rising temperatures, may extend the range of key mosquito vectors into previously unaffected areas, including Andean foothill and peri-urban zones, where *Aedes aegypti* and *Haemagogus* spp. can establish suitable habitats [9, 11]. This ecological disruption is further amplified by deforestation, habitat fragmentation, and the expansion of extractive activities, such as illegal logging, illicit crop cultivation, and artisanal mining, which destabilize local ecosystems, increase human contact with sylvatic vectors, and facilitate viral spillover into immunologically naïve populations [10, 12]. In the urban cycle, *Aedes aegypti* mediates human–mosquito–human transmission in densely populated settings and has historically sustained large-scale epidemics.

A third, increasingly recognized “ecotone” or peri-urban cycle operates in transitional zones between sylvatic and urban environments, precisely the ecological interface where expanding agricultural frontiers, deforestation, and peri-urban growth bring human communities into direct contact with sylvatic transmission dynamics [13]. Globalization compounds these dynamics by accelerating the conditions that enable the spread of disease [14]. While temperature and rainfall are primary determinants of YF transmission, influencing mosquito survival, biting behavior, and viral replication, they can paradoxically serve as outbreak catalysts [15] with subsequent spread into cooler, more humid urban environments underscoring the complexity of the disease’s epidemiological drivers [15].

Vaccination remains the only effective strategy for preventing YF outbreaks [8], with lifelong immunity after administration and an estimated efficacy of 97.9% [16, 17]. Furthermore, vaccination activities avert approximately 94 000 to 119 000 cases each year in Africa and South America [18]. An estimated 450 million vaccine doses will be required to reduce the global infection risk by 2026. In Colombia, routine vaccination has been estimated to be cost-effective, averting over 2200 non-fatal cases, 65 deaths, and 1365 disability-adjusted life-years during the interepidemic period between 1980 and 2002 [19]. However, vaccination coverage remains suboptimal, estimated at 65% nationally. Notably, vaccination in Colombia has traditionally been recommended for people living in or traveling to recognized endemic areas, where previous outbreaks have been documented [20].

Although YF has been extensively described from epidemiological and entomological perspectives, the detailed characterization of critically ill patients in contemporary outbreaks remains limited [14]. This study aims to characterize the clinical presentation, patterns of organ dysfunction, supportive interventions, and outcomes of critically ill adults with reverse transcription polymerase chain reaction (RT-PCR)–confirmed YF admitted to the national referral intensive care unit (ICU) in the Andean region of Colombia during the 2024–2025 outbreak.

## METHODS

### Study Overview

This prospective observational cohort study was conducted at the national referral hospital in Ibagué, Colombia, designated by the Colombian Ministry of Health to manage YF during the outbreak declared by Colombia's National Institute of Health (Instituto Nacional de Salud, INS*)* during 2024–2025, between March 2025 and September 2025, where all adults with confirmed YF cases were included. Clinical, laboratory, and therapeutic data were collected prospectively using standardized case report forms (CRFs) and electronic medical records. The primary objective was to characterize the clinical presentation, patterns of organ dysfunction, supportive interventions, and in-hospital outcomes and to explore factors associated with in-hospital mortality.

### Study Population

The cohort comprised all consecutive adults aged 18 years or older admitted to the ICU with laboratory-confirmed YF during the study period. Diagnosis was established by RT-PCR performed on blood samples in accordance with national reference laboratory protocols [21]. Quantitative viral load and cycle threshold values were not available for analysis.

### Definitions

Clinical definitions for suspected and confirmed YF cases were predefined according to PAHO/WHO and Colombian National Institute of Health surveillance guidelines. Suspected cases were based on compatible clinical presentation and epidemiological exposure, whereas confirmed cases required RT-PCR confirmation [6, 21]. Criteria for ICU admission and definitions of organ dysfunction were predefined in accordance with international guidelines and published clinical definitions and are summarized in Supplementary Tables 1 and 2 [22–30]. Shock was recorded using predefined clinical criteria; etiology was not prospectively adjudicated, and the available variables did not permit reliable retrospective classification into mutually exclusive hemodynamic phenotypes.

### Data Collection and Statistical Analysis

Clinical data were collected prospectively using the Research Electronic Data Capture (REDCap®) platform hosted on a secure institutional server at Universidad de La Sabana [31, 32], through the ISARIC/WHO Clinical Characterisation Protocol for Severe Emerging Infections [33, 34]. Data capture was conducted using a CRF based on ISARIC ARC [35], a structured, machine-readable library of standardized questions designed to support rapid and harmonized data collection across outbreaks.

The CRF used to characterize YF during this outbreak was adapted for this specific study context using ISARIC BRIDGE [36], a web-based platform that facilitates CRF design and customization. Demographic and epidemiological variables included age, sex, residence, occupation, recent travel, and self-reported prior YF vaccination. Vaccination documentation and timing of prior vaccination were recorded when available. Bacterial infection status was recorded prospectively; organism- and specimen-level data were not available in the analytical dataset.

Data analysis was performed in ISARIC VERTEX [37], a web-based application that allows analysis of ISARIC ARC-formatted data and generates tables and visualizations to support key research questions. VERTEX is part of the ISARIC Clinical Epidemiology Platform, developed to standardize and streamline data collection and rapid analysis in outbreak-related clinical research.

Categorical variables were summarized using frequencies and percentages and continuous variables using medians and interquartile ranges (IQRs). For the prespecified survival status comparisons in the original analysis, categorical variables were compared using the χ^2^ test and continuous variables using Student's t-test when normally distributed or the Mann–Whitney U test when non-normally distributed. Given the modest sample size and multiple comparisons, *P* values were interpreted as exploratory rather than confirmatory. Variables added descriptively in response to peer review were not subjected to additional hypothesis testing.

### Ethical Considerations

The study protocol and informed consent forms were reviewed by the Institutional Review Board/Independent Ethics Committee of Hospital Federico Lleras Acosta, Ibagué, Colombia (approval code 08), Universidad de La Sabana (MED-122-2025), and Clinica Universidad de La Sabana (Code: 20250504). Written informed consent was obtained from all participants or their legal representatives before enrollment. The study was conducted in accordance with the Declaration of Helsinki, applicable Colombian regulations 8430/1993, and institutional data protection standards. This study was reported in accordance with the Strengthening the Reporting of Observational Studies in Epidemiology (STROBE) statement; the completed STROBE checklist is provided in the Supplementary Data.

## RESULTS

During March and September 2025, 62 adults with RT-PCR–confirmed YF were identified at the national referral ICU in Ibagué, Tolima, in the Andean region of Colombia. One patient was excluded due to transfer to another center, resulting in 61 patients in the final analysis: 39 survivors and 22 non-survivors, corresponding to an in-hospital CFR of 36.1% (Figure 1). The geographic context of the outbreak and referral area is shown in Figure 2. Intensive care unit admissions peaked in April, when 25 patients were admitted, and declined progressively thereafter (Supplementary Figure 1). Most patients were referred from other municipalities (96.7%; 59/61), and previous YF vaccination was self-reported by 16.4% (10/61).

**Figure 1.**
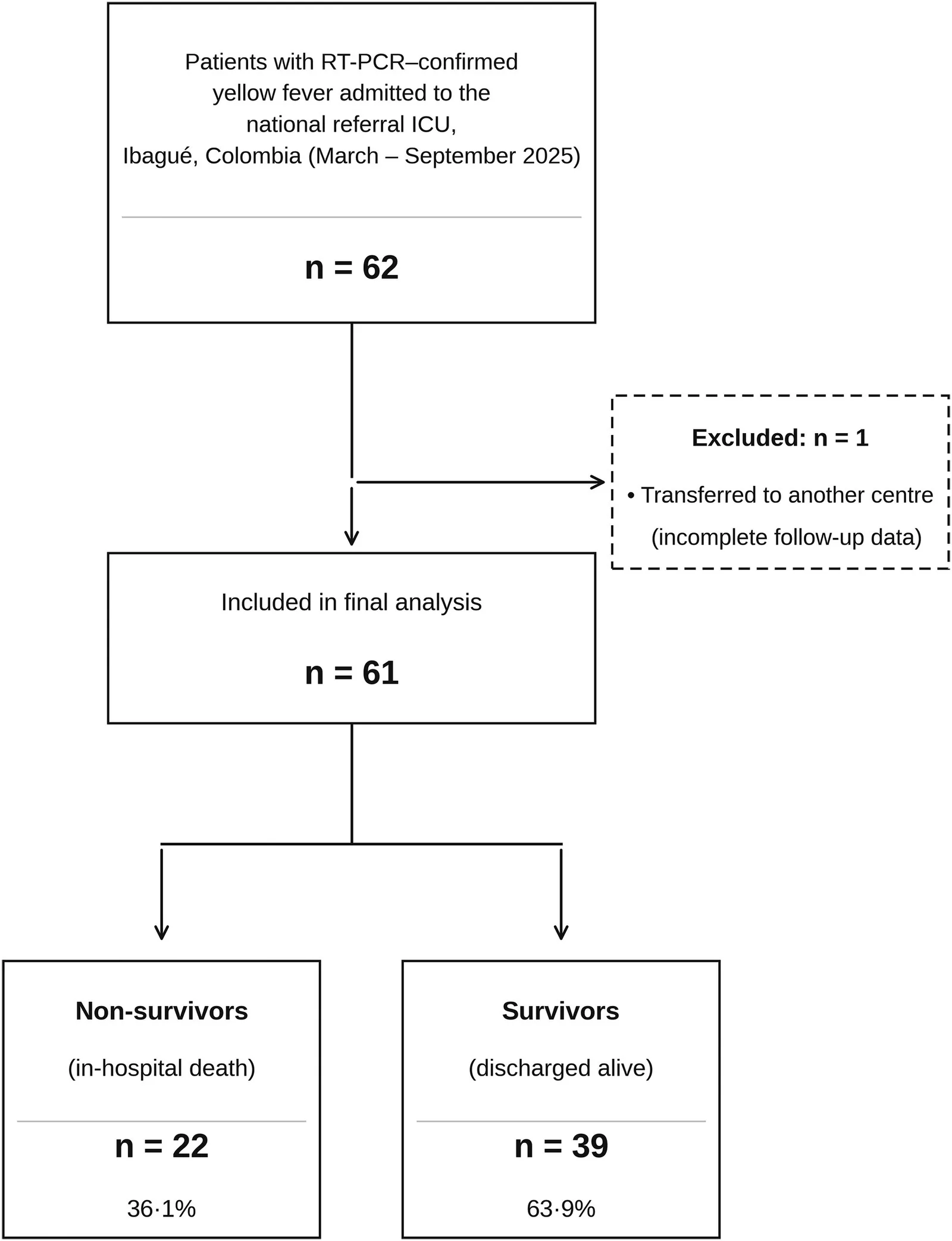
Flow diagram of patient inclusion. The flow diagram shows adults with reverse transcription polymerase chain reaction (RT-PCR)–confirmed yellow fever admitted to the national referral intensive care unit (ICU) in Ibagué, Colombia, from March through September 2025. Of 62 patients identified, one was excluded because of transfer to another center, leaving 61 patients in the final analysis: 22 non-survivors and 39 survivors. Abbreviations: ICU, intensive care unit; RT-PCR, reverse transcription polymerase chain reaction; YF, yellow fever.

**Figure 2.**
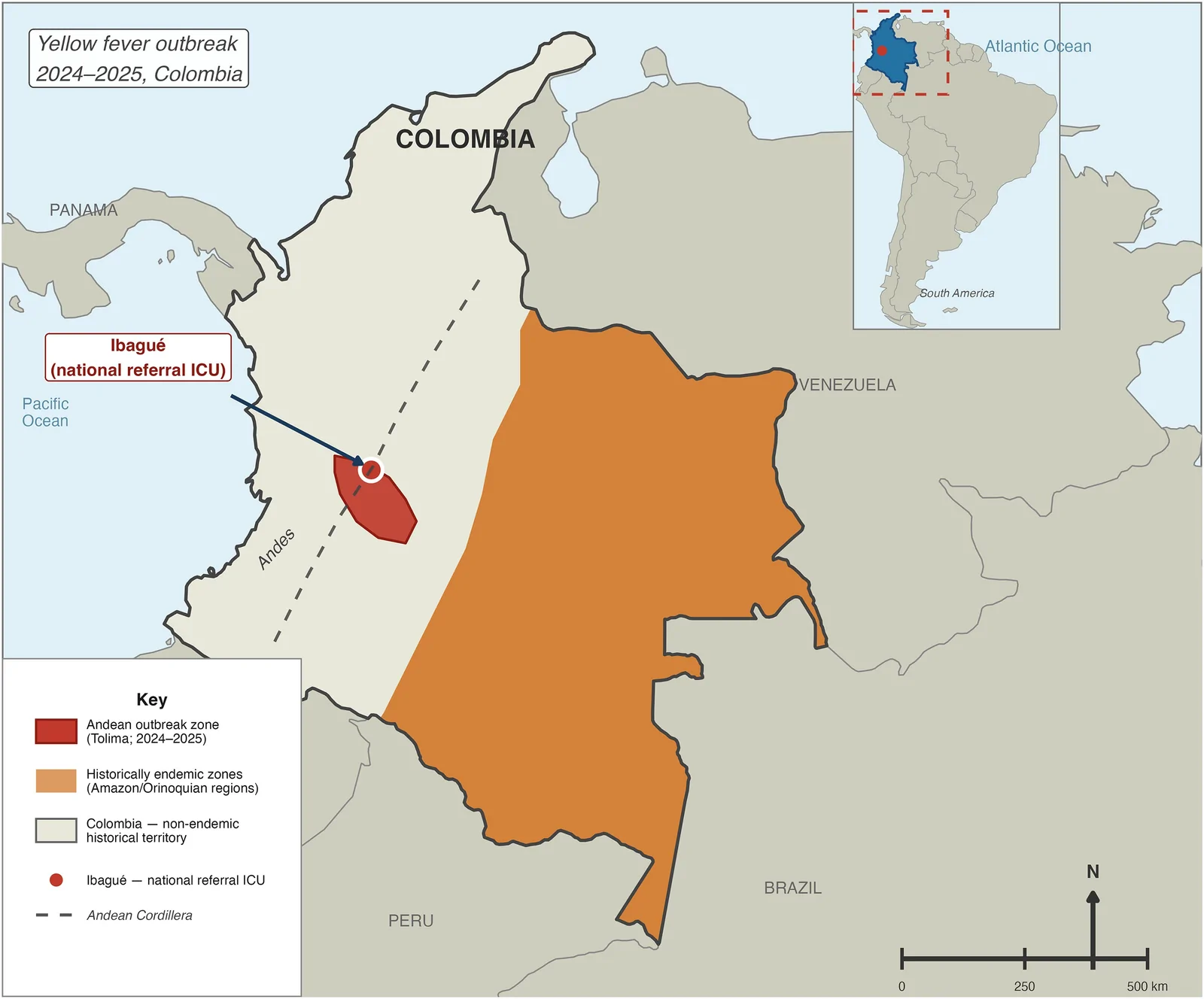
Geographic context of the 2024–2025 yellow fever outbreak in Colombia. The map shows the Andean outbreak zone in Tolima during the 2024–2025 yellow fever outbreak, the location of Ibagué as the national referral ICU, and the historically endemic Amazonian and Orinoquian regions. The figure highlights the occurrence of severe yellow fever requiring critical care outside Colombia's historically recognized endemic areas. Abbreviations: ICU, intensive care unit; YF, yellow fever.

### Clinical Presentation and Laboratory Findings at ICU Admission

The cohort was predominantly male (85.2%; 52/61), with a median age of 47 years (IQR 35–62). Median age was 58 years (IQR 35.5–69.8) among non-survivors and 45 years (IQR 34.0–59.5) among survivors; 90.9% (20/22) of non-survivors and 82.1% (32/39) of survivors were male. Outdoor agriculture, forestry, or fisheries occupations were recorded in 45.9% (28/61) and outdoor occupations involving animal contact in 11.5% (7/61). Fifty-nine patients (96.7%) resided outside the city of the referral center, while 7 (11.5%) reported travel outside their home region during the 14 days before symptom onset. These variables describe recorded epidemiological characteristics and do not establish the location or mechanism of infection. Comorbidities were uncommon; hypertension was documented in 13.1% (8/61) and diabetes mellitus in 6.6% (4/61). Presenting symptoms are shown in Table 1.

**Table 1.** Demographic, Epidemiological, Clinical, and Laboratory Characteristics at ICU Admission by Survival Status

Variable	All (n = 61)	Non-survivors (n = 22)	Survivors (n = 39)	*P* Value
Demographic and epidemiological characteristics				
Age, years, median (IQR)	47 (35–62)	58 (35.5–69.8)	45 (34.0–59.5)	—
Male sex, n (%)	52 (85.2)	20 (90.9)	32 (82.1)	—
Hypertension, n (%)	8 (13.1)	6 (27.3)	2 (5.1)	—
Diabetes mellitus, n (%)	4 (6.6)	3 (13.6)	1 (2.6)	—
Residence outside the referral-center city, n (%)	59 (96.7)	22 (100.0)	37 (94.9)	—
Travel outside home region within 14 d before symptom onset, n (%)	7 (11.5)	1 (4.5)	6 (15.4)	—
Occupation data available, n (%)	45 (73.8)	13 (59.1)	32 (82.1)	—
Recorded outdoor occupation, n (%)	35 (57.4)	10 (45.5)	25 (64.1)	—
Recorded agriculture/forestry/fisheries occupation, n (%)	28 (45.9)	7 (31.8)	21 (53.8)	—
Recorded outdoor occupation involving animal contact, n (%)	7 (11.5)	3 (13.6)	4 (10.3)	—
Self-reported prior YF vaccination, n (%)	10 (16.4)	3 (13.6)	7 (17.9)	—
Signs and symptoms at presentation, n (%)				
Fever, chills, or rigors	57 (93.4)	20 (90.9)	37 (94.9)	.951
Fatigue/malaise/lethargy	46 (75.4)	16 (72.7)	30 (76.9)	.955
Joint pain/arthralgia	39 (63.9)	12 (54.5)	27 (69.2)	.385
Muscle aches/myalgia	37 (60.7)	10 (45.5)	27 (69.2)	.121
Headache	32 (52.5)	8 (36.4)	24 (61.5)	.104
Abdominal pain	31 (50.8)	12 (54.5)	19 (48.7)	.865
Vomiting	23 (37.7)	8 (36.4)	15 (38.5)	1.000
Jaundice	23 (37.7)	9 (40.9)	14 (35.9)	.910
Diarrhea	18 (29.5)	4 (18.2)	14 (35.9)	.244
Bleeding/hemorrhage	8/60 (13.3)	5/21 (23.8)	3/39 (7.7)	.176
Laboratory findings at ICU admission, median (IQR)				
Platelets, ×10^9^/L	106 (67–143)	73 (52–106)	118 (92–180)	<.001
Prothrombin time, s	14.6 (11.8–19.3)	25.6 (18.5–45.4)	12.5 (11.6–14.7)	<.001
Activated partial thromboplastin time, s	38.5 (31.6–47.8)	47.9 (43.3–55.1)	34.0 (29.3–41.2)	<.001
INR	1.4 (1.1–1.9)	2.6 (1.8–5.1)	1.2 (1.1–1.4)	<.001
Fibrinogen, mg/dL	136 (72–186)	96 (54–142)	146 (114–221)	<.001
D-dimer, ng/mL	7757 (3537–11 791)	11 182 (7569–12 306)	4985 (1860–8938)	<.001
Total bilirubin, mg/dL	1.6 (0.6–4.2)	3.9 (2.2–5.7)	1.0 (0.5–2.4)	<.001
ALT, U/L	2110 (830–5906)	7333 (1862–9574)	1536 (467–3001)	<.001
AST, U/L	2687 (854–7640)	11 568 (3548–17 874)	1385 (268–3760)	<.001
Blood urea nitrogen, mg/dL	14.5 (10.8–32.0)	32.9 (21.2–48.3)	11.4 (10.2–17.4)	<.001
Creatinine, mg/dL	1.0 (0.8–1.6)	1.8 (1.2–5.3)	0.9 (0.8–1.1)	<.001
LDH, U/L	2088 (721–5625)	9374 (3207–11 993)	1240 (520–3273)	<.001

Data are n (%) unless otherwise specified. Continuous variables are presented as median (IQR). Occupation categories denote recorded characteristics; occupation data were available for 45/61 patients. Existing survival-group comparisons are exploratory. Variables added descriptively in response to peer review are shown without additional hypothesis testing (—).

Abbreviations: ALT, alanine aminotransferase; AST, aspartate aminotransferase; ICU, intensive care unit; INR, international normalized ratio; IQR, interquartile range; LDH, lactate dehydrogenase; YF, yellow fever.

In contrast, laboratory findings at ICU admission showed marked differences when stratified by survival status (Table 1). Compared with survivors, non-survivors had lower platelet counts (73 × 10^9^/L [IQR 52–106] vs 118 × 10^9^/L [92–180]; *P* < .001), longer prothrombin time (25.6 seconds [18.5–45.4] vs 12.5 seconds [11.6–14.7]; *P* < .001), longer activated partial thromboplastin time (47.9 seconds [43.3–55.1] vs 34.0 seconds [29.3–41.2]; *P* < .001), and higher INR (2.6 [1.8–5.1] versus 1.2 [1.1–1.4]; *P* < .001). Non-survivors also had lower fibrinogen (96 mg/dL [54–142] vs 146 mg/dL [114–221]; *P* < .001) and higher D-dimer concentrations (11 182 ng/mL [7569–12 306] vs 4985 ng/mL [1860–8938]; *P* < .001).

Markers of hepatic, renal, and tissue injury were also more pronounced among non-survivors. Total bilirubin was higher in non-survivors than survivors (3.9 mg/dL [2.2–5.7] vs 1.0 mg/dL [0.5–2.4]; *P* < .001), as were alanine aminotransferase (7333 U/L [1862–9574] vs 1536 U/L [467–3001]; *P* < .001), aspartate aminotransferase (11 568 U/L [3548–17 874] vs 1385 U/L [268–3760]; *P* < .001), blood urea nitrogen (32.9 mg/dL [21.2–48.3] vs 11.4 mg/dL [10.2–17.4]; *P* < .001), creatinine (1.8 mg/dL [1.2–5.3] vs 0.9 mg/dL [0.8–1.1]; *P* < .001), and lactate dehydrogenase (9374 U/L [3207–11 993] vs 1240 U/L [520–3273]; *P* < .001). Quantitative viral load and cycle threshold values were unavailable, precluding comparisons by survival status or vaccination history.

### In-hospital Complications and Outcomes

Non-survivors had higher frequencies of organ dysfunction and severe complications than survivors (Table 2). Severe liver disease occurred in 22/22 non-survivors and 19/39 survivors, hepatic encephalopathy in 19/19 and 8/18 patients with available data, acute kidney injury in 18/22 and 7/38, acute respiratory distress syndrome in 18/22 and 4/39, coagulation disorder or disseminated intravascular coagulation in 18/22 and 13/39, and shock in 20/22 and 3/39, respectively. Shock etiology was not systematically adjudicated and could not be reliably classified retrospectively from the available variables. Severe bleeding occurred in 11/22 non-survivors and 1/39 survivor. All 9 gastrointestinal bleeding events were clinically classified as upper gastrointestinal bleeding; endoscopy was not performed because of severe coagulopathy, so the anatomical source could not be further characterized. Seizures were recorded in 3/61 patients, all non-survivors and all with hepatic encephalopathy; specific antiseizure treatment was not systematically captured. Myocarditis was recorded in 1/61 patient; systematic quantitative troponin data were unavailable, precluding broader assessment of myocardial injury. Laboratory-confirmed bacterial infection was recorded in 10/61 patients; organism- and specimen-level microbiological data were not captured in the analytical dataset.

**Table 2.** In-hospital Complications and Clinical Outcomes by Survival Status

Variable	All (n = 61)	Non-survivors (n = 22)	Survivors (n = 39)	*P* Value
Organ complications, n (%)				
Acute hepatitis	56 (91.8)	22 (100.0)	34 (87.2)	.205
Severe liver disease (new onset)	41 (67.2)	22 (100.0)	19 (48.7)	<.001
Hepatic encephalopathy (any grade)	27/37 (73.0)	19/19 (100.0)	8/18 (44.4)	<.001
Acute kidney injury/acute renal failure	25/60 (41.7)	18/22 (81.8)	7/38 (18.4)	<.001
Acute respiratory distress syndrome	22 (36.1)	18 (81.8)	4 (10.3)	<.001
Coagulation disorder/DIC	31 (50.8)	18 (81.8)	13 (33.3)	<.001
Shock	23 (37.7)	20 (90.9)	3 (7.7)	<.001
Cardiac arrhythmia	12/60 (20.0)	11/21 (52.4)	1/39 (2.6)	<.001
Cardiac arrest	21 (34.4)	20 (90.9)	1 (2.6)	<.001
Severe bleeding	12 (19.7)	11 (50.0)	1 (2.6)	<.001
Upper gastrointestinal bleeding	9 (14.8)	9 (40.9)	0 (0.0)	<.001
Seizures	3 (4.9)	3 (13.6)	0 (0.0)	—
Myocarditis	1 (1.6)	1 (4.5)	0 (0.0)	—
Laboratory-confirmed bacterial infection	10 (16.4)	6 (27.3)	4 (10.3)	—
Pleural effusion	11 (18.0)	8 (36.4)	3 (7.7)	.014
In-hospital outcome, n (%)				
In-hospital mortality	22 (36.1)	—	—	—

Data are n (%) unless otherwise specified. Denominators are shown where data were missing. New reviewer-requested variables are reported descriptively without additional hypothesis testing (—). Shock etiology was not prospectively adjudicated. All 9 gastrointestinal bleeding events were clinically classified as upper gastrointestinal bleeding; endoscopy was not performed because of severe coagulopathy. Organism- and specimen-level data were not captured for laboratory-confirmed bacterial infection.

Abbreviations: AKI, acute kidney injury; ARDS, acute respiratory distress syndrome; DIC, disseminated intravascular coagulation.

### Therapeutic Interventions and Organ Support

Therapeutic interventions reflected the greater severity of illness among non-survivors (Table 3). Any blood product transfusion was more frequent in non-survivors than survivors (77.3% [17/22] vs 25.0% [9/36]; *P* < .001), including platelet transfusion (45.5% [10/22] vs 8.3% [3/36]; *P* = .003), fresh frozen plasma (54.5% [12/22] vs 13.9% [5/36]; *P* = .003), and packed red blood cells (27.3% [6/22] vs 0.0% [0/36]; *P* = .004). Cryoprecipitate use did not differ significantly between groups (22.7% [5/22] vs 5.6% [2/36]; *P* = .125). Corticosteroids were used more frequently in non-survivors than survivors (81.8% [18/22] vs 46.2% [18/39]; *P* = .014), as was N-acetylcysteine (68.2% [15/22] vs 35.9% [14/39]; *P* = .031). Antibiotic use was numerically higher among non-survivors but did not meet the predefined significance threshold (77.3% [17/22] vs 48.7% [19/39]; *P* = .057). Plasmapheresis was used in 45.9% of the cohort and did not differ significantly between non-survivors and survivors (54.5% [12/22] vs 41.0% [16/39]; *P* = .453), although duration was shorter among non-survivors (2.5 days [IQR 1.8–3.2] vs 3.0 days [3.0–4.0]; *P* < .001).

**Table 3.** Therapeutic Interventions and Organ Support by Survival Status

Variable	All (n = 61)	Non-survivors (n = 22)	Survivors (n = 39)	*P* Value
Blood product transfusion, n (%)				
Any blood product transfusion	26/58 (44.8)	17/22 (77.3)	9/36 (25.0)	<.001
Platelets	13/58 (22.4)	10/22 (45.5)	3/36 (8.3)	.003
Fresh frozen plasma	17/58 (29.3)	12/22 (54.5)	5/36 (13.9)	.003
Cryoprecipitate	7/58 (12.1)	5/22 (22.7)	2/36 (5.6)	.125
Packed red blood cells	6/58 (10.3)	6/22 (27.3)	0/36 (0.0)	.004
Pharmacological therapies and adjunctive treatment, n (%)				
Corticosteroids	36 (59.0)	18 (81.8)	18 (46.2)	.014
Antibiotics	36 (59.0)	17 (77.3)	19 (48.7)	.057
N-acetyl cysteine	29 (47.5)	15 (68.2)	14 (35.9)	.031
Plasmapheresis	28 (45.9)	12 (54.5)	16 (41.0)	.453
Duration, days, median (IQR)	3.0 (2.0–4.0)	2.5 (1.8–3.2)	3.0 (3.0–4.0)	<.001

Data are n (%) unless otherwise specified. Continuous variables are presented as median (IQR). Denominators are shown where data were missing. Between-group comparisons were exploratory and should be interpreted cautiously given the modest sample size.

Abbreviation: IQR, interquartile range.

### Exploratory Description According to Self-reported Prior YF Vaccination

Prior YF vaccination was self-reported by 10/61 patients (16.4%). None had documentary confirmation; the exact year, date, and age at vaccination were unavailable, although all reported vaccination more than 1 year before the current illness. No prior corticosteroid or other immunosuppressive therapy was recorded. Descriptive characteristics and outcomes according to self-reported vaccination history are provided in Supplementary Table 3. Severe liver disease occurred in 4/10 patients reporting prior vaccination and 37/51 without such a history; severe bleeding in 1/10 and 11/51; and in-hospital death in 3/10 and 19/51, respectively. These comparisons were exploratory and were not used to estimate vaccine effectiveness or to classify confirmed vaccine failure.

## DISCUSSION

In this cohort of adults admitted to the ICU during the 2024–2025 YF outbreak, 3 findings were notable. First, in-hospital mortality was 36.1%, and non-survivors had greater hepatic-coagulation derangement and renal and tissue injury at ICU admission. Second, non-survivors experienced frequent multisystem complications, including shock, acute kidney injury, acute respiratory distress syndrome, disseminated intravascular coagulation, and severe bleeding. Third, the outbreak occurred in an Andean region outside Colombia's historically recognized endemic areas, and 83.6% (51/61) did not report prior YF vaccination. Comparisons according to vaccination history were exploratory because prior vaccination was self-reported and could not be verified by vaccination records.

Our findings are consistent with previous hospital and ICU-based cohorts from Brazil, which have characterized severe YF as a high-mortality condition associated with profound hepatic injury, renal dysfunction, hemostatic derangement, and substantial critical-care support requirements [38–42]; however, this cohort contributes evidence from a non-Amazonian Andean setting, where low clinical familiarity, delayed recognition, and limited peripheral critical-care capacity may substantially amplify the consequences and progression of severe disease. Notably, presenting symptoms were largely nonspecific and did not discriminate between outcomes; early laboratory findings sharply differentiated survivors from non-survivors across markers of systemic damage (hepatocellular, coagulation, renal, and tissue).

The 2024–2025 YF outbreak in Tolima illustrates the limitations of relying on fixed historical endemic boundaries alone to guide prevention policy. The reemergence of YF in this Andean department, after nearly a century without confirmed cases and their institutional classification as low risk, closely mirrors the trajectory of the 2016–2018 Brazil outbreak, which swept through the Atlantic Forest region, historically considered non-endemic, causing over 2000 confirmed cases and approximately 750 deaths, primarily among unvaccinated adults in areas previously deemed safe [10, 43]. In both settings, transmission extended beyond historically recognized endemic boundaries, highlighting the limitations of static geographic risk classifications. The temporal and geographic similarities between these outbreaks support periodic reassessment of risk classifications using contemporary epidemiological, ecological, entomological, and vaccination-coverage data [12].

These findings highlight the limitations of restricting adult YF vaccination strategies to historically endemic zones without periodic risk reassessment. The risk classification of Tolima, maintained for decades based on the absence of 20th-century case records, may have contributed to substantial gaps in adult population immunity in a department with the ecological conditions, vector fauna, and non-human primate (NHP) reservoir species required for active sylvatic transmission [10, 44].

Vaccination in low-risk Colombian departments has been administered only to infants at 1 year of age since 2005, meaning that by the outbreak's onset, no adults under approximately 65 years in the affected municipalities had been systematically offered vaccination as adults [44]. The low frequency of reported prior vaccination among ICU-admitted patients (83.6% [51/61] did not report prior YF vaccination in the present cohort) is consistent with important historical gaps in vaccine coverage in the affected population [45]. The Colombian Ministry of Health has acknowledged this vulnerability in the emergency declaration of April 2025 [46], but the challenge of dynamically updating risk stratification models and extending vaccination campaigns to newly recognized at-risk territories requires sustained institutional investment rather than episodic emergency responses.

Limited systematic NHP and vector surveillance before the outbreak reduced opportunities for earlier detection. Non-human primate epizootics may precede human cases, providing a window for preventive action when captured by effective surveillance [47]. In Tolima, limited coordinated surveillance delayed recognition until human cases had spread across multiple municipalities [10]. The Colombian consensus and subsequent analyses have identified this gap as a surveillance priority [13, 39]. Experience from Brazil shows that genomic surveillance integrated with temporal and spatial data can identify viral dissemination pathways, supporting evaluation of a similar framework in Colombia combining YFV sequencing with epizootic, entomological, and vaccination-coverage data [48].

Further characterizing the severity profile of non-survivors, the coagulopathy observed in this cohort was consistent with a systemic consumptive phenotype [41, 42], evidenced by severe thrombocytopenia, prolonged clotting times, markedly elevated D-dimer, depleted fibrinogen, higher rates of DIC, and severe bleeding, which likely accounted for greater use of blood products among non-survivors (17/22, 77.3%) than survivors (9/36, 25.0%). Although plasmapheresis was frequently employed, its use did not differ significantly between survivors and non-survivors, precluding inference of treatment effect and underscoring the urgent need for prospective evaluation of adjunctive therapies and outbreak preparedness frameworks that encompass transfusion capacity, renal support, and coordinated access to critical care [49].

Beyond the clinical phenotype, this outbreak has important public health implications. The emergence of critically ill YF cases in Tolima and the Colombian Andes challenges static risk maps based on historically recognized endemic areas. Recent epidemiological analyses showed that the 2024–2025 outbreak was concentrated in the Magdalena Valley, particularly Tolima, previously considered low risk, with human and NHP infections [10]. In April 2025, the Colombian Ministry of Health declared a national health emergency, recognizing geographic expansion and the need to strengthen vaccination, surveillance, hospital alerts, ICU coordination, and referral pathways [46]. Prior YF vaccination was self-reported by only 10 patients and could not be verified by vaccination records; these descriptive findings should not be interpreted as estimates of vaccine effectiveness or as evidence of confirmed vaccine failure.

This study has several limitations. It was conducted at a single national referral center with a modest sample size; between-group comparisons and *P* values should therefore be interpreted as exploratory rather than confirmatory. The observational design precludes causal inference regarding supportive or adjunctive therapies. Vaccination history was self-reported in 10 patients without documentary confirmation, and the exact year or age at vaccination was unavailable. Quantitative viral load and systematically interpretable troponin measurements were not available. Shock etiology was not prospectively adjudicated, organism- and specimen-level microbiological data were not captured in the analytical dataset, specific antiseizure treatment was not systematically recorded, and autopsy or histopathological data were unavailable. These limitations constrain mechanistic interpretation but do not affect the prospective description of core clinical, laboratory, complication, intervention, and outcome variables in RT-PCR–confirmed cases.

In conclusion, severe YF during this Andean outbreak was characterized by hepatic-coagulation abnormalities, multisystem organ dysfunction, and substantial in-hospital mortality among critically ill adults. These findings describe the critical-care phenotype of YF in a Colombian Andean setting and support preparedness strategies that integrate clinical recognition, referral capacity, vaccination, and surveillance in areas where epidemiological risk is changing.

## Supplementary Material

ofag636_Supplementary_Data

## Supplementary Data

Supplementary materials are available at *Open Forum Infectious Diseases* online. Consisting of data provided by the authors to benefit the reader, the posted materials are not copyedited and are the sole responsibility of the authors, so questions or comments should be addressed to the corresponding authors.
