# Supplementary material for: Characterization of an Unusual Severe Yellow Fever Outbreak in the Colombian Andean Region
Source: Open Forum Infect Dis. 2026 Oct 21;13(10):ofag636. doi: 10.1093/ofid/ofag636 (PMC13684397; doi:10.1093/ofid/ofag636)
Supplement: Supplementary file 1 [file ofag636_supplementary_data.zip › Appendix_YF_2026.docx]

**Appendix**

**Supplementary Table 1. Case definition for yellow fever**

*Suspected and confirmed case definitions used for study eligibility.*

| **Case category** | **Definition** |
| --- | --- |
| Suspected case based on symptoms in an area with viral circulation | Patients who lived in, visited, or had epidemiological contact with areas recognised as zones of viral circulation and presented with:  1. Acute fever (temperature ≥38·5°C with sudden onset and duration <7 days); and  2. At least one of the following: headache, myalgia, nausea and/or vomiting, or abdominal pain. |
| Suspected case based on icterohaemorrhagic syndrome without a known viral circulation area | Any patient who, without a history of exposure to areas with viral circulation, presented abruptly with icteric syndrome associated with bleeding of any kind. |
| Confirmed case | Patient with positive reverse transcription-polymerase chain reaction. |

Clinical case definitions for suspected and confirmed yellow fever were based on Colombian National Health Institute surveillance guidance. Suspected cases were defined according to compatible clinical presentation and epidemiological exposure, whereas confirmed cases required positive reverse transcription polymerase chain reaction testing.

**Abbreviations:** RT-PCR = reverse transcription polymerase chain reaction.
**Source:** Adapted from Colombian National Health Institute yellow fever surveillance guidance.

**Supplementary Table 2. Criteria for admission to the intensive care unit**

*Liver injury and organ dysfunction criteria used to support ICU admission during the outbreak response.*

| **Category** | **Criteria** |
| --- | --- |
| Liver injury | 1. Elevation of liver enzymes (AST or ALT) >500 U/L.  2. Thrombocytopenia <100,000 platelets/mm³.  3. Prolonged INR >1·5.  4. Elevation of liver enzymes (AST or ALT) below 500 U/L with target organ injury. |
| Haemodynamic instability | Persistent hypotension after adequate fluid resuscitation requiring vasopressors to maintain MAP ≥65 mm Hg. |
| Acute respiratory failure | Failure of gas exchange manifested by PaO₂ <60 mm Hg or PaCO₂ ≥45 mm Hg with pH <7·35. |
| Acute kidney injury | Any elevation of serum creatinine ≥0·3 mg/dL within 48 h, or urine output <0·5 mL/kg/h for ≥6 h. |
| Acute neurological syndrome | Any Glasgow coma scale score <12, positive CAM-ICU delirium screen, or acute seizure (ILAE T1 >5 min). |
| Major active bleeding | Bleeding that is fatal, occurs in a critical site (intracranial, intraspinal, intraocular, pericardial, retroperitoneal, intra-articular, or intramuscular with compartment syndrome), causes a decrease in haemoglobin of ≥2 g/dL, or requires ≥2 units of RBC transfusion. |
| Metabolic disturbances | K⁺ ≥6·0–6·5 mmol/L or ≤2·5 mmol/L with or without arrhythmia or paralysis.  Na⁺ <120 mmol/L or ≥160 mmol/L with or without neurological symptoms.  Ca²⁺ <0·9 mmol/L.  pH ≤7·20 or ≥7·55. |

Criteria for intensive care unit admission included evidence of severe liver injury or acute organ dysfunction, defined according to international clinical guidelines. Patients meeting any criterion for liver injury or organ dysfunction were considered eligible for intensive care assessment during the outbreak response.

**Abbreviations:** AKI = acute kidney injury; ALT = alanine aminotransferase; AST = aspartate aminotransferase; CAM-ICU = Confusion Assessment Method for the Intensive Care Unit; Hb = haemoglobin; ICU = intensive care unit; ILAE = International League Against Epilepsy; INR = international normalised ratio; MAP = mean arterial pressure; PaCO₂ = partial pressure of arterial carbon dioxide; PaO₂ = partial pressure of arterial oxygen; RBC = red blood cells.
**Source:** Adapted from international guidelines for sepsis, acute respiratory failure, acute kidney injury, delirium, seizures, major bleeding, and metabolic disturbances.
