# Supplementary material for: Characterization of an Unusual Severe Yellow Fever Outbreak in the Colombian Andean Region
Source: Open Forum Infect Dis. 2026 Oct 21;13(10):ofag636. doi: 10.1093/ofid/ofag636 (PMC13684397; doi:10.1093/ofid/ofag636)
Supplement: Supplementary file 1 [file ofag636_supplementary_data.zip › Supplementary_YF_2026_Lancet-2.pdf]

Supplementary Material

Characterisation of an Unusual Severe Yellow Fever Outbreak in the Colombian Andean Region

The Lancet Regional Health – Americas

Table of Contents

Item	Description	Page
Supplementary Table 1	Case definitions for yellow fever	2
Supplementary Table 2	Criteria for ICU admission	3
STROBE Checklist	Reporting checklist for cohort studies	4–7

Supplementary Table 1. Case definitions for yellow fever

Suspected and confirmed case definitions used for study eligibility.

Case category	Definition
**Suspected case based on symptoms in an area with viral circulation**	Patients who lived in, visited, or had epidemiological contact with areas recognised as zones of viral circulation and presented with: (1) acute fever (temperature $\geq 38 \cdot 5$°C with sudden onset and duration <7 days); and (2) at least one of the following: headache, myalgia, nausea and/or vomiting, or abdominal pain.
**Suspected case based on icterohaemorrhagic syndrome without a known viral circulation area**	Any patient who, without a history of exposure to areas with viral circulation, presented abruptly with icteric syndrome associated with bleeding of any kind.
**Confirmed case**	Patient with positive reverse transcription-polymerase chain reaction (RT-PCR).

Abbreviations: RT-PCR = reverse transcription polymerase chain reaction.

Source: Adapted from Colombian National Health Institute yellow fever surveillance guidance.

Supplementary Table 2. Criteria for admission to the intensive care unit

Liver injury and organ dysfunction criteria used to support ICU admission during the outbreak response.

Category	Criteria
**Liver injury**	1. Elevation of liver enzymes (AST or ALT) >500 U/L.<br>2. Thrombocytopenia <100,000 platelets/mm^3.<br>3. Prolonged INR >1·5.<br>4. Elevation of liver enzymes (AST or ALT) below 500 U/L with target organ injury.
**Haemodynamic instability**	Persistent hypotension after adequate fluid resuscitation requiring vasopressors to maintain MAP ≥65 mm Hg.
**Acute respiratory failure**	Failure of gas exchange manifested by PaO_2 <60 mm Hg or $PaCO_2$ ≥45 mm Hg with pH <7·35.
**Acute kidney injury**	Any elevation of serum creatinine ≥0·3 mg/dL within 48 h, or urine output <0·5 mL/kg/h for ≥6 h.
**Acute neurological syndrome**	Any Glasgow coma scale score <12, positive CAM-ICU delirium screen, or acute seizure (ILAE T1 >5 min).
**Major active bleeding**	Bleeding that is fatal, occurs in a critical site (intracranial, intraspinal, intraocular, pericardial, retroperitoneal, intra-articular, or intramuscular with compartment syndrome), causes a decrease in haemoglobin of ≥2 g/dL, or requires ≥2 units of RBC transfusion.
**Metabolic disturbances**	K^+ ≥6·0–6·5 mmol/L or ≤2·5 mmol/L with or without arrhythmia or paralysis.<br>Na^+ <120 mmol/L or ≥160 mmol/L with or without neurological symptoms.<br>Ca^{2+} <0·9 mmol/L. pH ≤7·20 or ≥7·55.

Abbreviations: AKI = acute kidney injury; ALT = alanine aminotransferase; AST = aspartate aminotransferase; CAM-ICU = Confusion Assessment Method for the Intensive Care Unit; ICU = intensive care unit; ILAE = International League Against Epilepsy; INR = international normalised ratio; MAP = mean arterial pressure; $PaCO_2$ = partial pressure of arterial carbon dioxide; PaO_2 = partial pressure of arterial oxygen; RBC = red blood cells.

Source: Adapted from international guidelines for sepsis, acute respiratory failure, acute kidney injury, delirium, seizures, major bleeding, and metabolic disturbances.

STROBE Checklist — Cohort Study

Strengthening the Reporting of Observational Studies in Epidemiology (STROBE) Statement: checklist of items that should be included in reports of cohort studies.

Manuscript: Characterisation of an Unusual Severe Yellow Fever Outbreak in the Colombian Andean Region. *The Lancet Regional Health – Americas*, 2026.

Section / Topic	Item No.	Recommendation	Reported on page No.
Title and abstract	1	(a) Indicate the study's design with a commonly used term in the title or the abstract.<br>(b) Provide in the abstract an informative and balanced summary of what was done and what was found.	Title page; Summary (p. 1–2)
Background / rationale	2	Explain the scientific background and rationale for the investigation being reported.	Introduction (p. 3–4)
Objectives	3	State specific objectives, including any prespecified hypotheses.	Introduction, last paragraph (p. 4)
Study design	4	Present key elements of study design early in the paper.	Methods – Study overview (p. 4)
Setting	5	Describe the setting, locations, and relevant dates, including periods of recruitment, exposure, follow-up, and data collection.	Methods – Study overview (p. 4)
Participants	6	(a) Give the eligibility criteria, and the sources and methods of selection of participants. Describe methods of follow-up.<br>(b) For matched studies, give matching criteria and number of exposed and unexposed.	Methods – Study population (p. 4–5)
Variables	7	Clearly define all outcomes, exposures, predictors, potential confounders, and effect modifiers. Give diagnostic criteria, if applicable.	Methods – Definitions (p. 5); Suppl. Tables 1–2
Data sources / measurement	8*	For each variable of interest, give sources of data and details of methods of assessment. Describe comparability of assessment methods if there is more than one group.	Methods – Data collection and statistical analysis (p. 5–6)
Bias	9	Describe any efforts to address potential sources of bias.	Discussion – Limitations (p. 10–11)
Study size	10	Explain how the study size was arrived at.	Methods – Study overview (p. 4); Results (p. 6)
Quantitative variables	11	Explain how quantitative variables were handled in the analyses. If applicable, describe which groupings were chosen and why.	Methods – Statistical analysis (p. 6)
Statistical methods	12	(a) Describe all statistical methods, including those used to control for confounding.<br>(b) Describe any methods used to examine subgroups and interactions.<br>(c) Explain how missing data were addressed.<br>(d) If applicable, explain how loss to follow-up was addressed.<br>(e) Describe any sensitivity analyses.	Methods – Statistical analysis (p. 6)
Participants	13*	(a) Report numbers of individuals at each stage of study.■<br>(b) Give reasons for non-participation at each stage.<br>(c) Consider use of a flow diagram.	Results (p. 7); Figure 1 (flow diagram)

Section / Topic	Item No.	Recommendation	Reported on page No.
Descriptive data	14*	(a) Give characteristics of study participants and information on exposures and potential confounders.<br>(b) Indicate number of participants with missing data for each variable of interest.<br>(c) Summarise follow-up time.	Results – Clinical presentation (p. 7); Table 1
Outcome data	15*	Report numbers of outcome events or summary measures over time.	Results – Complications and outcomes (p. 8); Tables 2–3
Main results	16	(a) Give unadjusted estimates and, if applicable, confounder-adjusted estimates and their precision.<br>(b) Report category boundaries when continuous variables were categorised.<br>(c) If relevant, consider translating estimates of relative risk into absolute risk.	Results (p. 7–10); Tables 1–3
Other analyses	17	Report other analyses done — eg analyses of subgroups and interactions, and sensitivity analyses.	Results – Vaccinated vs unvaccinated (p. 9–10)
Key results	18	Summarise key results with reference to study objectives.	Discussion (p. 10)
Limitations	19	Discuss limitations of the study, taking into account sources of potential bias or imprecision. Discuss both direction and magnitude of any potential bias.	Discussion – Limitations (p. 10–11)
Interpretation	20	Give a cautious overall interpretation of results considering objectives, limitations, multiplicity of analyses, results from similar studies, and other relevant evidence.	Discussion (p. 10–11)
Generalisability	21	Discuss the generalisability (external validity) of the study results.	Discussion – Limitations (p. 10–11)
Funding	22	Give the source of funding and the role of the funders for the present study.	Summary – Funding; Acknowledgements

* Give information separately for exposed and unexposed groups in cohort studies.

Note: The STROBE checklist is best used in conjunction with the Explanation and Elaboration article (freely available at www.strobe-statement.org).
